# Influence of being videotaped on the prevalence effect during visual search

**DOI:** 10.3389/fpsyg.2015.00583

**Published:** 2015-05-06

**Authors:** Yuki Miyazaki

**Affiliations:** ^1^School of Psychology, Chukyo University, Nagoya, Japan; ^2^Research Institute for Multimodal Sensory Science, Kanagawa University, Yokohama, Japan

**Keywords:** visual search, video monitoring, evaluation apprehension, motivation, mere effort, low-prevalence effect, signal detection theory

## Abstract

Video monitoring modifies the task performance of those who are being monitored. The current study aims to prevent rare target-detection failures during visual search through the use of video monitoring. Targets are sometimes missed when their prevalence during visual search is extremely low (e.g., in airport baggage screenings). Participants performed a visual search in which they were required to discern the presence of a tool in the midst of other objects. The participants were monitored via video cameras as they performed the task in one session (the videotaped condition), and they performed the same task in another session without being monitored (the non-videotaped condition). The results showed that fewer miss errors occurred in the videotaped condition, regardless of target prevalence. It appears that the decrease in misses in the video monitoring condition resulted from a shift in criterion location. Video monitoring is considered useful in inducing accurate scanning. It is possible that the potential for evaluation involved in being observed motivates the participants to perform well and is related to the shift in criterion.

## Introduction

It is well known that task performance is altered by being observed by someone else (i.e., social facilitation or inhibition; Bond and Titus, 1983; Guerin, 1993, for reviews). According to classical views, observer presence or mere social presence enhances the emission of dominant responses (Zajonc and Sales, 1966; Cottrell et al., 1968). Performance is facilitated when the dominant response is appropriate (mostly for simple tasks or well-trained tasks), while it is inhibited when the response is inappropriate. The emission of dominant responses is also enhanced by video monitoring as well as the physical presence of observers (e.g., Henchy and Glass, 1968; Cohen, 1979). Video cameras have traditionally been used to manipulate observer presence (e.g., in a memory recall task, Geen, 1973; in a vigilance task, Putz, 1975), and it has been demonstrated that video monitoring has a similar effect to observer presence.

Although the underlying mechanism of the effects of observer presence and video monitoring remains debatable, an influential theory proposes that the potential evaluative aspect implicated in the observation modifies the participant’s behavior. Most participants tend to become concerned about how they look when they perform a task in front of observers or video cameras. The apprehension about being evaluated increases the dominant response (Cottrell, 1968). This evaluation apprehension theory has been supported by previous studies that used a manipulation of observer presence or video monitoring (e.g., Cottrell et al., 1968; Cohen, 1979).

Recently, Harkins (2006) and his colleagues (McFall et al., 2009) suggested the mere effort account. In contrast to evaluation apprehension theory (Cottrell, 1968), the mere effort account argues that the potential for evaluation motivates participants to perform well, which enhances the production of the dominant response. However, if the dominant response is recognized as being an incorrect response, motivation leads to a correction of the mistake (although the correction requires cognitive control and sufficient time). In sum, the difference between the mere effort account and evaluation apprehension theory lies in the mediation of motivational aspects and top-down correction for an incorrect dominant response.

The mere effort account is mostly consistent with the results of studies that investigate the effect of observer presence using the Stroop task (Sharma et al., 2010) and visual search tasks. It has been demonstrated that participants perform visual search (in which they judge the presence of a target from among distractors, and in which 50% of the trials are target-present trials) more accurately if they are being observed or videotaped (Miyazaki, 2013). In support of signal detection theory (Macmillan and Creelman, 2005), it has also been found that the criterion location was negatively shifted if participants were being observed or videotaped during a visual search task. The negative direction represents a liberal response bias, which is the tendency to respond “target present.” In general, participants are more prone to making miss errors (i.e., “target absent” responses in target-present trials) than false-alarm errors (i.e., “target present” responses in target-absent trials) in the typical visual search task. These findings are explained as participants changing the decision criterion to inhibit miss errors based on the social motivation to perform well. Such a shift in decision criterion has also been found in collaborative visual search in pairs (in which it is supposed that the participants are monitoring each other) in comparison to visual search whereby each participant independently searches for a target (Malcolmson et al., 2007). Overall, during visual search, participants seem to attempt to reduce their mistakes in order to perform well in front of video cameras or others by modifying their decision criterion.

Individuals who engage in activities that involve a visual search of the environment, such as during airport baggage checks or medical cancer screenings, sometimes miss targets, such as dangerous substances or tumors (Kundel, 1982; Egglin and Feinstein, 1996; Evans et al., 2011). In these situations, target prevalence, which is the frequency of targets presented during visual search, is extremely low (e.g., Fenton et al., 2007). Recent laboratory studies using an artificial baggage screening task have highlighted similar results, wherein low target prevalence led to high miss rates during visual search. This effect is called the low-prevalence effect (Wolfe et al., 2005, 2007; Fleck and Mitroff, 2007; Van Wert et al., 2009; Godwin et al., 2010a,b; Lau and Huang, 2010; Wolfe and Van Wert, 2010; Ishibashi et al., 2012). The low-prevalence effect is neither due to decreasing vigilance in regards to the targets, nor one’s unfamiliarity with them (Wolfe et al., 2007). Instead, it is attributed to the extremely early termination of search in target-absent trials (Wolfe et al., 2005; Rich et al., 2008; Wolfe and Van Wert, 2010), a criterion shift toward a conservative position (see also Gur et al., 2007; Wolfe et al., 2007; Wolfe and Van Wert, 2010), and/or motor-execution errors (Fleck and Mitroff, 2007).

The solution to eliminate the low-prevalence effect is a debatable issue. Wolfe et al. (2007) hypothesized that a criterion shift toward a conservative position (which is the tendency to respond “target absent”) plays a crucial role in the low-prevalence effect, and they attempted to calibrate the criterion location toward a neutral position. In the visual search task, they inserted a high-prevalence block (40 trials) with incorrect/correct feedback about the last response prior to each low-prevalence visual search block (200 trials), which participants completed without feedback. By maintaining the neutral criterion of high-prevalence search during the low-prevalence block, the miss rates were decreased. The intervention of a high-prevalence block is effective in preventing the low-prevalence effect; however, it may be difficult to implement in daily screenings. For example, the airport staff engaging in baggage screening would not be able to take the time to perform frequent high-prevalence searches.

In a different approach to the problem, Fleck and Mitroff (2007) reported that the low-prevalence effect was eliminated in correctable searches. For low-prevalence searches, the participants pressed the same key assigned to the target-absent response repeatedly. The authors expected a strong motor response bias to be formed by repetition and believed this would contribute to the low-prevalence effect. For this reason, they provided opportunities for their participants to correct their answer after the key press. The correction option decreased the miss rates. However, more recently it was found that the correctable search had only a marginal effect on the low-prevalence effect when the task difficulty was high (Van Wert et al., 2009; Kunar et al., 2010). Although many additional approaches have been explored, such as the use of color or spatial cues (see Russell and Kunar, 2012), the integration of fake feedback (see Schwark et al., 2012), and the manipulation of expected reward (see Navalpakkam et al., 2009), the best approach to solving the low-prevalence effect is still unknown.

The purpose of the present research was to inhibit the low-prevalence effect through the video monitoring effect. The approach here was to prevent the shift in criterion toward a conservative position (e.g., Wolfe et al., 2007). As the evidence indicates that being observed during search leads to shifts in criterion to avoid mistakes (Malcolmson et al., 2007; Miyazaki, 2013), it is possible that the decision criterion is neutralized (i.e., shifted toward a liberal position) and the low-prevalence effect is inhibited by video monitoring.

In the present study, the participants performed an artificial baggage screening task and were required to judge the presence of a tool from among common objects, such as toys, fruit, clothing, and birds (see Wolfe et al., 2005). The participants performed the task whilst being monitored via video cameras in one session (the videotaped condition), and performed the task alone and unwatched in another session (the non-videotaped condition). Three visual search conditions related to target prevalence were used: low (2%), mid (10%), and high (50%). It was expected that participants’ miss rates would be lower in the videotaped condition than in the non-videotaped condition regardless of the target prevalence if video monitoring encourages a more liberal response strategy during visual search.

## Materials and Methods

### Participants

One hundred graduate and undergraduate students (65 females and 35 males; Mean = 19.39 years, SD = 1.20) were paid to participate (1,000 yen/h). None of the participants were aware of the purpose of the study, and all had normal or corrected-to-normal eyesight. This study was approved by the Research Ethics Committee of the School of Psychology in Chukyo University. Written informed consent was obtained from all participants.

### Apparatus

**FIGURE 1:**
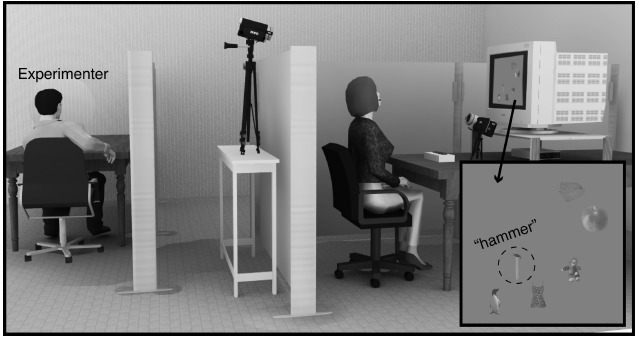
**Illustrations of the experimental environments and an example of the search items.** In the videotaped condition, study subjects were recorded by two digital video cameras. In the non-videotaped condition, the digital video cameras were removed. The subjects were required to judge the presence of a tool from among other category items (toy, fruit, clothing, and bird). Note: a central crosshair and visual noise were superimposed on the search display.

The experiment was controlled by MATLAB 2012a (Math-Works, Natick, MA, USA) with Psychophysics Toolbox extensions (Brainard, 1997; Pelli, 1997; Kleiner et al., 2007) on a desktop computer (Apple Mac mini). The visual stimuli were projected on a 22-inch cathode ray tube (CRT) monitor (Mitsubishi RDF223G; monitor resolution = 1280 × 960 pixels, refresh rate = 90 Hz). Reaction times (RTs) were measured using a response time box (Li et al., 2010). Two digital video cameras mounted on tripod stands were used during the videotaped condition. One of the cameras was placed in front of the participant, and the other was placed behind him/her (Figure 1). The front camera did not interfere with the participant’s view of the display.

### Stimuli

The search display was composed of a target item and/or distracter items, a light-gray central crosshair, and a mid-gray background. In addition, visual noise (noise density: 40%) consisting of random black pixels was superimposed on the search display. The luminance values of light gray, mid-gray, and black were 17.14, 7.48, and 4.47 cd/m^2^ respectively. The target and distracter items were photorealistic objects drawn from commercial image collections (Hemera Photo-Objects). Six target items were selected from the tool category: hammer, saw, plier, ax, drill, and wrench. The distracter items were selected from four categories, which were “toy” (puzzle, blocks, kite, doll, yo-yo, and ball), “fruit” (grape, peach, apple, pear, cherry, and orange), “clothing” (shirt, dress, shorts, socks, pants, and vest), and “bird” (penguin, duck, owl, eagle, parrot, and chicken). To gage familiarity with the target items, the researcher had the participants confirm knowledge of each of these before beginning the experiment.

The target and distracter items were approximately the same size (3.63° × 3.63°; 120 × 120 pixels) and were converted into semi-transparent portable network graphics (PNG) images by a graphics editor. Each item was randomly located in a cell of an invisible 5 × 5 grid (18.01° × 18.01°) and was randomly shifted between ±1.51° horizontally and vertically from the center of the cell in which it was positioned. The central crosshair was 0.30°, and there were six items on the search display.

### Experimental Design

The author employed a 2 × 3 × 2 within-participants design. The variables of interest were the following: being videotaped or not during visual search task (videotaped and non-videotaped conditions); target prevalence in the visual search task (low-, mid-, and high-prevalence conditions); and target presence in the visual search task (target-present and target-absent trials).

The low-prevalence condition comprised 500 trials, of which 2% were target-present trials. The mid- and high-prevalence conditions comprised 100 trials, of which 10 and 50%, respectively, were target-present trials. The 700 test trials were separated into seven blocks: five were low-prevalence, one was mid-prevalence, and the remaining one was high-prevalence. Half of the participants performed the test trials in the following order: five low-prevalence blocks, one mid-prevalence block, and one high-prevalence block. The remaining participants performed everything in the reverse order. Before starting each block, participants read a message, which appeared on the display, informing them about the target prevalence condition. They also performed 25 practice trials prior to the test trials, when the target prevalence condition was switched. In the target-present trials, the search items were composed of one target item and five distracter items. In the target-absent trials, six distracter items were presented in the display.

### Procedure

Upon arrival at the laboratory, each participant was greeted by a male experimenter and provided with an informed consent form. The experimenter explained the visual search task, involving an artificial baggage-screening situation consisting of 2 sessions × 775 trials, to the participants. The experimenter also told the participant that his/her behavior was being videotaped in only one session (the videotaped condition) and that their behavior would be analyzed via video clips by an unknown individual after the experiment. In the second session, each participant performed the visual search task alone, and the digital video cameras were removed (the non-videotaped condition). The experimenter stayed at a separate booth in both conditions. Half of the participants first underwent the videotaped condition, while the remaining participants performed the task in the non-videotaped condition.

The experiment was conducted in a well-lit room. Each trial started with a crosshair presented at the center of the display for 500 ms. The search display appeared next and remained until a response was made or for 10,000 ms. Visual feedback (“Correct” or “Wrong”) was provided on the display after response selection or after 10,000 ms (the time limit). The next trial began after a 500 ms blank display. The participants were required to judge whether there was a tool among the search items. They were required to respond, as quickly and accurately as possible, by pressing one of two appropriately labeled buttons (“target present” or “target absent”). After every 100 trials, they were prompted to take a break.

The participants wore headphones playing white noise to suppress ambient sounds in the experimental room. They were instructed to keep their viewing distance at 60 cm from the CRT display, although they were free to move their heads. Their eye movements were not constrained.

## Results

Errors were divided into two types: miss errors and false-alarm errors. Three participants were excluded from the analysis due to their aberrant task performance. One participant made 57.82% false-alarm errors, another participant made 27.25% premature (<150 ms) response errors, and the third participant made miss errors more than 3 SDs from the group mean. After the data of the three participants were removed, trials of lower than 150 ms or higher than 10,000 ms (0.19% of all trials) and the practice trials were removed from statistical analyses.

In preliminary analyses, neither the main effects of the order of video monitoring condition, nor of the order of target prevalence condition, were identified as significant using analysis of variance (ANOVA). To enhance the statistical power, these between-participants factors were collapsed in the following statistical analyses.

### Accuracy

Most importantly, a 2 (video monitoring) × 3 (target prevalence) ANOVA for the mean of the miss rates after arc-sine transformation revealed a significant main effect of video monitoring [*F*(1,96) = 4.040, *p* = 0.047, ${\text{η}}_{\text{p}}^{\text{2}}$ = 0.040], indicating that the miss rates in the videotaped condition were lower than those in the non-videotaped condition (Table 1). A significant main effect of target prevalence was also revealed [*F*(2,192) = 155.684, *p* < 0.001, ${\text{η}}_{\text{p}}^{\text{2}}$ = 0.619]. Multiple comparisons using Holm’s method for target prevalence identified significant differences between the three conditions in miss rates [low- vs mid-prevalence conditions, *t*(96) = 9.215, *p* < 0.001, *r* = 0.685; low- vs high-prevalence conditions, *t*(96) = 17.757, *p* < 0.001, *r* = 0.876; mid- vs high-prevalence conditions, *t*(96) = 8.301, *p* < 0.001, *r* = 0.646]. These results indicated that decreasing the target prevalence produced much higher miss rates. No significant interaction between video monitoring and target prevalence was shown [*F*(2,192) = 0.356, *p* = 0.701, ${\text{η}}_{\text{p}}^{\text{2}}$ = 0.004]. In sum, the low-prevalence effect was exhibited under both the videotaped and non-videotaped conditions. However, the video monitoring effect brought a decrease in misses regardless of the target prevalence.

**TABLE 1:** **Miss rate (in %), false alarm rate (in %), *d*′, and *c* as a function of target prevalence and video monitoring conditions**.

	**Low-prevalence—2%**		**Mid-prevalence—10%**		**High-prevalence—50%**		**Main effects of video monitoring**	
	**Videotaped**	**Non-videotaped**	**Videotaped**	**Non-videotaped**	**Videotaped**	**Non-videotaped**	**F(1,96)**	**${\text{η}}_{\text{p}}^{\text{2}}$**
%Miss	30.72	33.40	16.46	18.97	5.95	6.31	4.04*	0.04
	(2.05)	(2.07)	(1.52)	(1.49)	(0.52)	(0.46)		
%FA	0.22	0.13	0.45	0.37	2.43	2.73	0.19	>0.01
	(0.11)	(0.02)	(0.10)	(0.08)	(0.33)	(0.40)		
*d*′	3.54	3.45	3.49	3.40	3.71	3.63	3.41	0.03
	(0.07)	(0.07)	(0.06)	(0.06)	(0.06)	(0.06)		
*c*	1.19	1.24	0.70	0.76	0.17	0.19	4.56*	0.05
	(0.03)	(0.03)	(0.03)	(0.02)	(0.02)	(0.02)		

Standard errors are in parentheses. *p<0.05.

There was a significant main effect of target prevalence [*F*(2,192) = 96.811, *p* < 0.001, ${\text{η}}_{\text{p}}^{\text{2}}$ = 0.502] on the mean false-alarm rate after arc-sine transformation. Multiple comparisons by Holm’s method revealed significant differences between the three conditions in false-alarm rates [low- vs mid-prevalence conditions, *t*(96) = 2.481, *p* = 0.015, *r* = 0.245; low- vs high-prevalence conditions, *t*(96) = 10.613, *p* < 0.001, *r* = 0.735; mid- vs high-prevalence conditions, *t*(96) = 10.085, *p* < 0.001, *r* = 0.717]. In contrast to misses, increasing the target prevalence produced many more false alarms. Wolfe et al. (2007) reported higher false-alarm rates in the high-prevalence condition compared to the low-prevalence condition. The current study replicated this finding. Furthermore, the main effect of video monitoring, and interaction between video monitoring and target prevalence, did not reach significance [*F*(1,96) = 0.188, *p* = 0.666, ${\text{η}}_{\text{p}}^{\text{2}}$ = 0.002; *F*(2,192) = 0.999, *p* = 0.370, ${\text{η}}_{\text{p}}^{\text{2}}$ = 0.010], indicating that the false-alarm rate was not influenced by video monitoring.

### Sensitivity and Criterion

Where necessary, the proportions of 1.0 and 0 (i.e., perfect accuracy) were adjusted to avoid infinite values (Macmillan and Creelman, 2005). The parameters of *d′* (sensitivity) and *c* (criterion location) were calculated for each participant and each condition. Although only parametric measures in the signal detection theory were reported here, the trends of the results were similar to those of the non-parametric measures.

A 2 (video monitoring) × 3 (target prevalence) ANOVA for *d′* revealed a significant main effect of target prevalence [*F*(2,192) = 10.392, *p* < 0.001, ${\text{η}}_{\text{p}}^{\text{2}}$ = 0.098]. Multiple comparison using Holm’s method for target prevalence identified significant differences between *d′* in the low-prevalence condition and high-prevalence condition [*t*(96) = 3.148, *p* = 0.002, *r* = 0.306]. There was also a significant difference between *d′* in the mid-prevalence condition and high-prevalence condition [*t*(96) = 4.362, *p* < 0.001, *r* = 0.407]. The main effect of video monitoring, and the interaction between video monitoring and target prevalence, did not reach significance [*F*(1,96) = 3.410, *p* = 0.068, ${\text{η}}_{\text{p}}^{\text{2}}$ = 0.034; *F*(2,192) = 0.0003, *p* = 0.999, ${\text{η}}_{\text{p}}^{\text{2}}$ < 0.001], indicating that sensitivity was not changed by the video monitoring.

Regarding *c*, ANOVA revealed significant main effects of video monitoring [*F*(1,96) = 4.561, *p* = 0.035, ${\text{η}}_{\text{p}}^{\text{2}}$ = 0.045] and target prevalence [*F*(2,192) = 711.747, *p* < 0.001, ${\text{η}}_{\text{p}}^{\text{2}}$ = 0.881]. No significant interaction was found between the two factors [*F*(2,192) = 0.584, *p* = 0.559, ${\text{η}}_{\text{p}}^{\text{2}}$ = 0.006]. Multiple comparison by Holm’s method for target prevalence identified significant differences between the three conditions for *c* [low- vs mid-prevalence conditions, *t*(96) = 18.497, *p* < 0.001, *r* = 0.884; low- vs high-prevalence conditions, *t*(96) = 33.455, *p* < 0.001, *r* = 0.960; mid- vs high-prevalence conditions, *t*(96) = 22.169, *p* < 0.001, *r* = 0.915]. In summary, criterion location was positively (conservatively) shifted by decreasing the target prevalence in both the videotaped and non-videotaped conditions, which concurs with Wolfe et al.’s (2007) findings. More importantly, however, the criterion location was calibrated and was negatively (liberally) shifted by the video monitoring, regardless of target prevalence.

### Reaction Time

Reaction times from trials in which an incorrect response was made were excluded from the following analyses. A 2 (video monitoring) × 3 (target prevalence) × 2 (target presence) ANOVA for RT did not show a significant effect of video monitoring [*F*(1,96) = 0.091, *p* = 0.764, ${\text{η}}_{\text{p}}^{\text{2}}$ = 0.001]. It did reveal significant effects of target prevalence [*F*(2,192) = 8.609, *p* < 0.001, ${\text{η}}_{\text{p}}^{\text{2}}$ = 0.082] and target presence [*F*(1,96) = 7.737, *p* = 0.007, ${\text{η}}_{\text{p}}^{\text{2}}$ = 0.075], and a significant interaction between target prevalence and target presence [*F*(2,192) = 434.617, *p* < 0.001, ${\text{η}}_{\text{p}}^{\text{2}}$ = 0.819]. No other significant interaction was found. Simple effects tests for the interaction between target prevalence and target presence found that, in the high-prevalence condition, RT in the target-absent trials was slower than in the target-present trials [*F*(1,96) = 154.069, *p* < 0.001, ${\text{η}}_{\text{p}}^{\text{2}}$ = 0.616]. In contrast, in the low- and mid-prevalence conditions, the RT in the target-absent condition was faster than in the target-present condition [*F*(1,96) = 193.224, *p* < 0.001, ${\text{η}}_{\text{p}}^{\text{2}}$ = 0.668; *F*(1,96) = 13.070, *p* < 0.001, ${\text{η}}_{\text{p}}^{\text{2}}$ = 0.120]. These trends are consistent with the findings of previous studies (e.g., Wolfe et al., 2005).

To examine whether misses in the low-prevalence condition were derived from a speed-accuracy trade-off (i.e., whether misses increased with speed), with regard to the target-absent trials, the mean RT in the low-prevalence condition was subtracted from that in the high-prevalence condition for each participant and for each video monitoring condition. The difference represented the magnitude of acceleration in the low-prevalence condition relative to the high-prevalence condition (see Kunar et al., 2010; Russell and Kunar, 2012). If the elevation of the miss rates in the low-prevalence condition was due to the speed-accuracy trade-off, then the difference in RT and the miss rates (after arc-sine transformation) in the low-prevalence condition would be positively correlated. However, no significant correlations were shown in either the videotaped or non-videotaped conditions [*t*(95) = –0.346, *p* = 0.730, *r* = –0.036; *t*(95) = 1.419, *p* = 0.159, *r* = 0.144]. Likewise, in the mid-prevalence condition, the mean RT was subtracted from that observed in the high-prevalence condition. There were no significant correlations in either the videotaped or non-videotaped conditions [*t*(95) = 1.017, *p* = 0.312, *r* = 0.104; *t*(95) = 0.128, *p* = 0.899, *r* = 0.013]. Although it is necessary to be cautious with the explanations of the results due to the insignificant effects, these results showed that the low-prevalence effect was not affected by a speed-accuracy trade-off, which supports the results of previous studies (e.g., Wolfe et al., 2007).

## Discussion

The goal of this study was to determine whether the low-prevalence effect is inhibited by video monitoring. As expected, participants searched more accurately when they were being observed via video cameras, regardless of target prevalence. The results of the present study offer new support for the idea that video monitoring is effective in preventing miss errors during visual search, regardless of target prevalence. Importantly, video monitoring also alters the criterion location during visual search; the criterion was shifted in a more liberal direction. Such a criterion modification has also been suggested by Wolfe et al. (2007) to prevent the low-prevalence effect. It is thought that the reduction of miss errors derives from the shift in criterion.

How does video monitoring influence the shift in the criterion? The results could be explained by the mere effort account (e.g., Harkins, 2006), arguing that the potential for evaluation motivates the participants to perform well. The motivation contributes to an increase of dominant response, and elicits correction of the mistake when the dominant response is inappropriate. The author believes that the potential for evaluation derived from the video monitoring is related to the shift in criterion via the mediation of motivation. In the visual search task of the present study, miss errors were more salient than false-alarm errors, and the participants easily perceived the errors from the correct/incorrect feedback about the latest response. It is considered that the participants modified the criterion toward a more liberal position to inhibit miss errors, which is a straightforward strategy to use to avoid mistakes and to display an acceptable performance. On the other hand, the current results cannot be explained by the evaluation apprehension theory (Cottrell, 1968). If the theory is correct, the dominant target-absent responses would be enhanced by the video monitoring (particularly in the low-prevalence condition), resulting in misses being increased and the decision criterion being shifted to a more conservative position. In fact, the opposite results were observed in the present study with regard to misses and the decision criterion. Although it was indeed unclear whether the participants believed that they could be evaluated via the video monitoring, the current findings were highly consistent with the mere effort account. In future studies, it is necessary to check the belief about the evaluation to confirm the relationship between potential for evaluation and video monitoring.

Note that the present study succeeded in only reducing, but not eliminating, the low-prevalence effect. The effect size of video monitoring on miss rates was small (${\text{η}}_{\text{p}}^{\text{2}}$ = 0.040, see Table 1). More studies are required in future to investigate the method for enhancing the video monitoring effect. For example, the effect size might be increased by manipulating the number of digital video cameras used for monitoring, or their installation positions. Although the effect size remains a debatable issue, the video monitoring method has one advantage beyond other methods (Fleck and Mitroff, 2007; Wolfe et al., 2007). In addition to preventing the failure to detect targets, video monitoring provides an opportunity for conducting a post-analysis based on the recorded video clips if a miss (or near miss) unfortunately happens during screening. It would provide the clues needed to investigate the cause of the miss.

**FIGURE 2:**
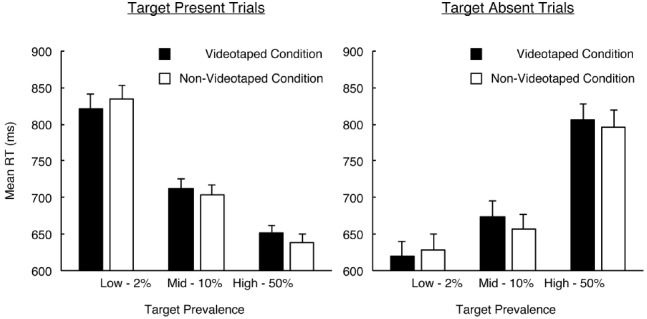
**Mean reaction time (RT; ms) as a function of video monitoring condition × target prevalence condition × target presence in visual searches.** Error bars represent standard errors.

Although RTs in the videotaped condition were generally slower than those in the non-videotaped condition (see Figure 2), a statistically significant difference between the two conditions was not found. According to the multiple decision model (Wolfe and Van Wert, 2010), the participants use two decision processes during visual search. One is the decision criterion determined by signal detection theory (i.e., *c*) and the other is a decision threshold related to the termination of the visual search. The participants first make judgments for a target-present response based on the decision criterion. Target-present responses are made if a perceived stimulus exceeds the criterion. If not, the participants continue to search. While the visual search is continuing, a quitting signal to terminate the search is gradually accumulated. The visual search is terminated early (i.e., the participants respond “target-absent”) regardless of the target detection if the quitting signal reaches the decision threshold. The performance in low-prevalence visual search is predicted by the multiple decision model. The decision criterion is more conservative and the decision threshold becomes lower in low-prevalence visual search, resulting in an increase of miss errors. Once again, the present study found that the significant effect of video monitoring was shown only in the *c*, and a significant effect was not shown in the RTs. Taken together, it is possible that video monitoring only influences the decision criterion in terms of *c*, but does not affect the decision threshold in terms of RTs.

Wolfe et al. (2007) reported that sensitivity did not change as a function of target prevalence (in fact, in some experiments, sensitivity was slightly higher in the low-prevalence condition than in the high-prevalence condition). However, in the present study, sensitivity in the low- and mid-prevalence conditions was lower than in the high-prevalence condition. Indeed, the decline in sensitivity in the low-prevalence condition, relative to the high-prevalence condition, has also been reported in previous studies (Van Wert et al., 2009, Experiment 1A). A little sensitivity fluctuation might be involved in the low-prevalence effect. However, the results indicate that sensitivity fluctuation is extremely small compared to that of the criterion (see Table 1). Therefore, the author believes that the present results support the hypothesis that the low-prevalence effect is mainly caused by the shift in criterion toward a more conservative position (e.g., Wolfe et al., 2007).

A number of limitations of the present study should be highlighted. First, the number of items in the search display was not manipulated. It is not clear whether the effect of video monitoring would influence visual search in which the number of search items is either smaller or larger than six. Second, task difficulty was not manipulated. Indeed, with regard to the effect of the correctable search (Fleck and Mitroff, 2007), effect sizes were determined on the basis of task difficulty (Van Wert et al., 2009; Kunar et al., 2010). Future studies should manipulate both the number of search items and task difficulty. This manipulation would show conclusively whether the influence of video monitoring on the low-prevalence effect depends on the stimulus context.

The present study raises the possibility that video monitoring is an effective approach to preventing misses during visual search. Due to the lack of interaction between the effects of video monitoring and target prevalence, it contributes to preventing failure to detect targets in some types of visual search. In real-world tasks where the detection of targets is vital, directly monitoring participants may boost their performance, although it remains unclear whether expert searchers are susceptible to these effects. In summary, people need to behave carefully under some visual search situations in order to avoid a tragic accident, such as a fatal car accident due to a driver overlooking a pedestrian or an airline hijacking resulting from overlooking dangerous substances during baggage screening. The present study raises the possibility that the use of video monitoring systems could be a viable new approach for encouraging more careful behaviors in life-critical visual search situations.

### Conflict of Interest Statement

The author declares that the research was conducted in the absence of any commercial or financial relationships that could be construed as a potential conflict of interest.
